# Supplementary material for: Zinc inhibits the voltage-gated proton channel HCNL1
Source: Biophys J. 2024 Aug 28;123(24):4256–65. doi: 10.1016/j.bpj.2024.08.018 (PMC11700363; doi:10.1016/j.bpj.2024.08.018)
Supplement: Document S1. Figures S1–S3 [file mmc1.pdf]

Biophysical Journal, Volume *123*

Supplemental information

Zinc inhibits the voltage-gated proton channel HCNL1

Makoto F. Kuwabara, Joschua Klemptner, Julia Muth, Emilia De Martino, Dominik Oliver, and Thomas K. Berger

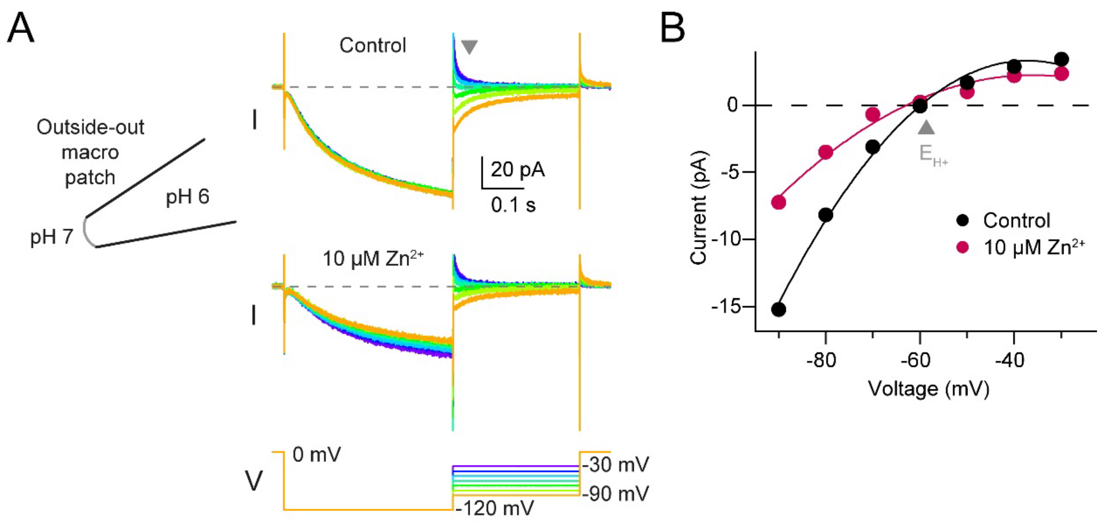

FIGURE S1. Zn^{2+} does not permeate through HCNL1. (A) Current traces of an excised outside-out macro patch containing HCNL1 channels in response to hyperpolarizing voltage steps in the absence (Control) or presence of 10 μM Zn^{2+} applied to the bath. (B) Current–voltage relationships of the tail currents in panel (A). E_{H+} is the Nernst potential for protons (-59.2 mV at 25° C and intra- and extracellular pH of 6 and 7, respectively), indicated by the gray triangle. If HCNL1 conducted Zn^{2+}, the tail current would reverse its sign at less negative potentials. Instead, there is a slight shift towards more negative potentials (control, V_{rev} = -62.3 ± 1.6 mV; 10 μM Zn^{2+}, V_{rev} = -64.6 ± 1.2 mV, $n_{patches}$ = 4). Zeros crossing were determined by fitting the data to quadratic functions.

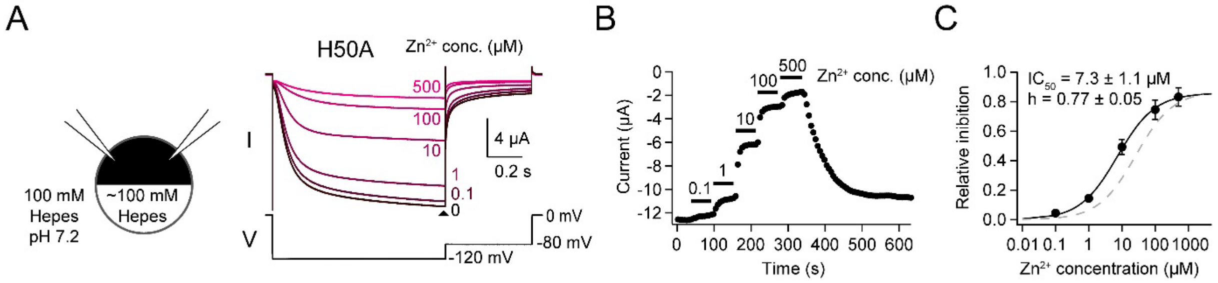

FIGURE S2. Similar to WT, HCNL1-H50A is inhibited by extracellular Zn^{2+}. (A) Left, cartoon of the TEVC recording configuration. Prior to recording, the pH buffer capacity was increased by an injection of HEPES. Right, representative HCNL1-H50A-mediated inward currents in response to a hyperpolarizing voltage step in the presence or absence of various Zn^{2+} concentrations applied to the recording chamber. (B) Steady-state current amplitudes during Zn^{2+} application, derived from the data of panel (A) at the time point indicated by the triangle. (C) Concentration-response curve determined from the relative current inhibition ($n_{oocytes}$ = 6). The extrapolated maximal inhibition was 85.9 ± 6.0%. The concentration response of WT (same data as in Figure 1D) is depicted as a gray dashed curve for comparison. Data are represented as the mean ± SD.

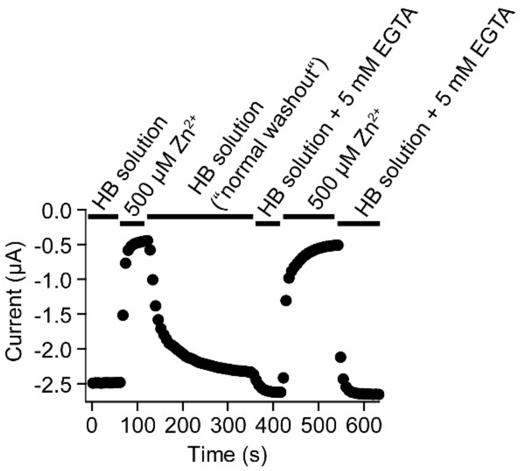

FIGURE S3. Extracellular application of EGTA improves current recovery after Zn^{2+} washout. Steady-state current amplitudes of an HCNL1-expressing oocyte before and after extracellular application of 500 µM Zn^{2+} in the absence ("normal washout") or presence of 5 mM EGTA during washout. Current recovery after washout of Zn^{2+} was 89.7 ± 6.8% in the absence of EGTA and 99.6 ± 1.7% in the presence of EGTA ($n_{Oocytes}$ = 6).
