# Supplementary material for: Zinc inhibits the voltage-gated proton channel HCNL1
Source: Biophys J. 2024 Aug 28;123(24):4256–65. doi: 10.1016/j.bpj.2024.08.018 (PMC11700363; doi:10.1016/j.bpj.2024.08.018)
Supplement: Document S2. Article plus supporting material [file mmc2.pdf]

Biophysical Journal
Article

Biophysical Society

Zinc inhibits the voltage-gated proton channel HCNL1

Makoto F. Kuwabara,[1] Joschua Klemptner,[1] Julia Muth,[1] Emilia De Martino,[1] Dominik Oliver,[1] and Thomas K. Berger[1,*]
[1]Department of Neurophysiology, Institute of Physiology and Pathophysiology, Philipps University Marburg, Marburg, Germany

ABSTRACT Voltage-gated ion channels allow ion flux across biological membranes in response to changes in the membrane potential. HCNL1 is a recently discovered voltage-gated ion channel that selectively conducts protons through its voltage-sensing domain (VSD), reminiscent of the well-studied depolarization-activated Hv1 proton channel. However, HCNL1 is activated by hyperpolarization, allowing the influx of protons, which leads to an intracellular acidification in zebrafish sperm. Zinc ions (Zn^{2+}) are important cofactors in many proteins and essential for sperm physiology. Proton channels such as Hv1 and Otopetrin1 are inhibited by Zn^{2+}. We investigated the effect of Zn^{2+} on heterologously expressed HCNL1 channels using electrophysiological and fluorometric techniques. Extracellular Zn^{2+} inhibits HCNL1 currents with an apparent half-maximal inhibition (IC_{50}) of 26 μM. Zn^{2+} slows voltage-dependent current kinetics, shifts the voltage-dependent activation to more negative potentials, and alters hyperpolarization-induced conformational changes of the voltage sensor. Our data suggest that extracellular Zn^{2+} inhibits HCNL1 currents by multiple mechanisms, including modulation of channel gating. Two histidine residues located at the extracellular side of the VSD might weakly contribute to Zn^{2+} coordination: mutants with either histidine replaced with alanine show modest shifts of the IC_{50} values to higher concentrations. Interestingly, Zn^{2+} inhibits HCNL1 at even lower concentrations from the intracellular side ($IC_{50} \approx 0.5$ μM). A histidine residue at the intracellular end of S1 (position 50) is important for Zn^{2+} binding: much higher Zn^{2+} concentrations are required to inhibit the mutant HCNL1-H50A ($IC_{50} \approx 106$ μM). We anticipate that Zn^{2+} will be a useful ion to study the structure-function relationship of HCNL1 as well as the physiological role of HCNL1 in zebrafish sperm.

SIGNIFICANCE Two voltage-gated proton channels have been described: the depolarization-activated Hv1 channel and the recently discovered hyperpolarization-activated HCNL1 channel. Both channels harbor an unusual ion-permeation pathway: protons permeate through the channels' voltage-sensing domain. Here, we discover that HCNL1, like Hv1, is sensitive to Zn^{2+}, a trace element important for spermatogenesis and male fertility in many species across phyla. HCNL1 is inhibited by Zn^{2+} from the extra- and intracellular side. HCNL1 is expressed in the plasma membrane of the head of zebrafish sperm, suggesting that Zn^{2+} might also play a role in the physiology of zebrafish sperm.

INTRODUCTION

Voltage-gated ion channels (VGICs) control the flux of ions across biological membranes which is important for electrical signaling in cells. Classical VGICs show a stereotypical and modular architecture: four subunits (or repeats) assemble to form a tetrameric (or pseudotetrameric) channel. Each subunit usually contains a voltage-sensing domain (VSD) and a pore domain (PD). The VSD has four transmembrane spanning segments (S1–S4), and the PD has two segments (S5–S6) that are connected via one or two short pore helices (Fig. 1 *A*). Each PD contributes one-quarter of the central pore, which usually contains the ion-permeation pathway. S4 contains several charged amino acid residues (mainly arginines) at every third position and functions as the main voltage sensor that operates a gate in the central pore. The selectivity filter in the pore determines the preferred permeating ion species; K^+, Na^+, and Ca^{2+} channels are often severalfold more selective for their respective preferred ion species over other ions (1).

The first discovered VGIC selective for protons, Hv1 (encoded by the gene HVCN1), revealed that proton channels are similar to classical VGICs yet also very different (2,3). Hv1 forms dimers (4–6) and each subunit contains only a

Submitted May 31, 2024, and accepted for publication August 23, 2024.
*Correspondence: thomas.berger@uni-marburg.de
Editor: Thomas DeCoursey.
https://doi.org/10.1016/j.bpj.2024.08.018
© 2024 The Author(s). Published by Elsevier Inc. on behalf of Biophysical Society.
This is an open access article under the CC BY license (http://creativecommons.org/licenses/by/4.0/).

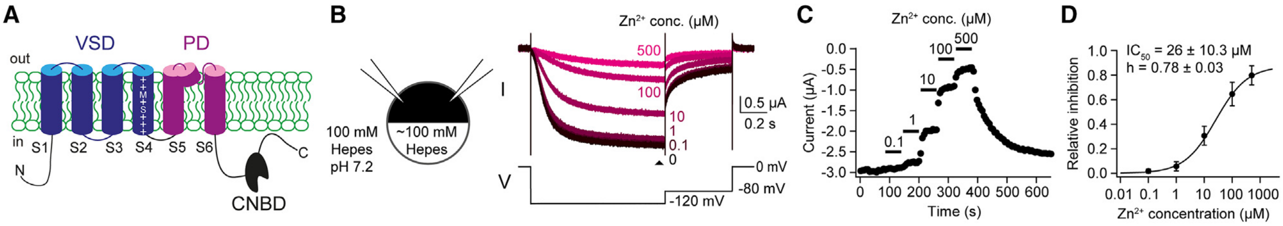

FIGURE 1 HCNL1 is inhibited by extracellular Zn^{2+}. (*A*) Cartoon of one subunit of the HCNL1 proton channel, consisting of a voltage-sensing domain, a pore domain, and an intracellular cyclic nucleotide-binding domain (CNBD). (*B*) Left: cartoon of the TEVC recording configuration. Before recording, the pH buffer capacity was increased by an injection of HEPES. Right: representative HCNL1-mediated inward currents in response to a hyperpolarizing voltage step in the presence or absence of various Zn^{2+} concentrations applied to the recording chamber. (*C*) Steady-state current amplitudes during Zn^{2+} application, derived from the data of (*B*) at the time point indicated by the triangle. (*D*) Concentration-response curve determined from the relative current inhibition ($n_{oocytes}$ = 10). Data are represented as the mean ± SD.

domain homologous to a VSD (hence also called voltage sensor domain-only protein). Each VSD contains its own, separate ion-permeation pathway (6), yet gating between the VSDs is cooperative (7,8). Activated by depolarization, Hv1 opens and allows proton conduction through its VSDs with an exquisitely high selectivity: protons are preferred over other cations by over a millionfold (9). The mechanism of proton permeation is not well understood; whether an aqueous proton-permeation pathway forms during conduction is debated (10,11). Hv1 is found across phyla from unicellular organisms to mammals and, in humans, is expressed in a variety of cells, including immune cells such as neutrophils (12), lung epithelial cells (13), and sperm (14). In human sperm, Hv1 has been suggested to be inhibited following ejaculation by high Zn^{2+} concentrations in the seminal plasma. Zn^{2+} is an allosteric modulator of Hv1, slowing activation and shifting activation to more positive membrane potentials (15). Following dilution of Zn^{2+} in the female reproductive tract, Hv1 has been suggested to contribute to the control of intracellular pH that in turn influences intracellular Ca^{2+} concentrations and thereby the sperm's swimming pattern (14).

We recently identified a second VGIC selective for protons: HCNL1 (16). HCNL1 is an unusual member of the family of hyperpolarization-activated, cyclic-nucleotide gated (HCN) ion channels. Canonical HCN channels are known as so-called pacemaker channels that open upon hyperpolarization and carry a Na^+ inward current to depolarize the cell (17–19). In contrast, HCNL1 conducts protons with a similarly high selectivity as Hv1. Stunning parallels between HCNL1 and Hv1 extend to the mechanism of proton conduction: HCNL1, like Hv1, harbors the proton-permeation pathway in its VSDs. The PD of HCNL1 is nonconducting. In both channels, proton conduction can be blocked or reduced by introducing an arginine residue at a position in register with the regularly spaced basic residues in VSD segment S4 (6,16,20–22). Of note, Hv1 is not only gated by voltage but also by the pH gradient across the membrane (2,3,23). It is not known if HCNL1 is also gated by the pH gradient. Interestingly, expression of HCNL1 is restricted to sperm and might be activated during spawning of zebrafish sperm (16).

Here, we study the inhibition of zebrafish HCNL1 by Zn^{2+}. Using electrophysiological and fluorescence-optical techniques, we show that Zn^{2+} inhibits HCNL1 from the extra- and also from the intracellular side. Our results suggest that HCNL1 current inhibition relies on multiple mechanism and is partially mediated by Zn^{2+} interfering with the voltage-dependent gating of HCNL1.

MATERIALS AND METHODS

Ethical approval

X. laevis oocytes were harvested from our own colony. Frogs were housed according to the German law of animal protection and the district veterinary office. Parts of an ovary were surgically obtained from frogs anesthetized in phosphate buffer containing 0.16% 3-aminobenzozate methanesulfonate salt. The surgery followed standard procedures and was carried out in accordance with the relevant guidelines and regulations with the approval (no. V9/2023) of the local authority of the state Hesse (Regierungspräsidium Gießen).

DNA constructs and expression in *X. laevis* oocytes

DNA constructs were made using standard molecular biological techniques and were confirmed by DNA sequencing. Single point mutations were introduced by primer mismatch. The cDNA encoding the zebrafish (*Danio rerio*) HCNL1 channel (accession no. QKM75727) was used in the pGEMHE vector for expression in *X. laevis* oocytes. All constructs contained a C-terminal HA-tag. RNA was in vitro transcribed from NheI-linearized DNA using the HiScribe T7 ARCA mRNA kit (New England Biolabs, Ipswich, MA). *X. laevis* oocytes were injected with 50 nL RNA (0.25–0.7 μg/μL) and incubated at 13–17°C for 1–5 days in ND96 medium containing: 96 mM NaCl, 2 mM KCl, 1.8 mM $CaCl_2$, 1 mM $MgCl_2$, 10 4-(2-hydroxyethyl) piperazine-1-ethanesulfonic acid (HEPES), and 5 mM Na-pyruvate, supplemented with 100 mg/L gentamicin and adjusted to pH 7.5 with NaOH.

Electrophysiological recordings

Two hours before two-electrode voltage clamp (TEVC) or voltage-clamp fluorometry (VCF) recordings, oocytes were injected with 50 nL of 1 M

HEPES (pH 7) to increase the pH buffer capacity of the oocyte (7). This way, the intracellular pH is stabilized during proton flux across the membrane. Right after injection, oocytes were incubated in a high buffer (HB) solution (7) containing: 88 mM NaCl, 1 mM KCl, 1 mM $MgCl_2$, 1 mM $CaCl_2$, and 100 mM HEPES (pH 7.2). During all TEVC and VCF recordings, oocytes were perfused in the HB recording solution. Pipettes for electrophysiological recordings were pulled from borosilicate capillaries (Hilgenberg, Malsfeld, Germany) using a DMZ puller (Zeitz Instruments, Martinsried, Germany). For TEVC recordings, the pipette solution contained 3 M KCl. For excised inside-out macropatch recordings (Figs. 6 and 7), both bath solution and pipette solution contained: 100 mM HEPES, 5 mM tetraethylammonium chloride (TEA-Cl), 30 mM methanesulfonic acid (MS), and 5 mM ethylene glycol tetraacetic acid (EGTA), adjusted to pH 7.0 with tetraethylammonium hydroxide (TEA-OH). For excised outside-out macropatch recordings (Fig. S1), the pipette solution contained: 100 mM HEPES, 5 mM TEA-Cl, 30 mM MS, and 5 mM EGTA, adjusted to pH 6.0 with TEA-OH. The tips of patch pipettes were polished using a Micro Forge (MF-830, Narishige, Tokyo, Japan) and coated with liquid paraffin (Merck, Darmstadt, Germany). The inner pipette diameter was 8–30 μm, giving an initial pipette resistance of 0.7–1.5 MΩ with the used pipette solutions. The reference electrode was connected to the bath solution via an agar bridge containing 3 M KCl. All experiments were conducted at room temperature (21–25°C). Chemicals were purchased from Sigma-Aldrich (St. Louis, MO), Merck, Fluka (Charlotte, NC), Thermo Fisher Scientific (Waltham, MA), or Carl Roth (Karlsruhe, Germany). The Zn^{2+}-containing solutions were prepared by progressive dilutions of a stock solution containing 500 mM $ZnCl_2$ and 10 mM HCl. All solutions were prepared from bidistilled, Milli-Q (Merck) filtered water.

TEVC recordings

TEVC recordings were performed with a TEC-10CX TEVC amplifier (npi electronic, Tamm, Germany) connected to a PC via an ITC-1600 data acquisition board (HEKA Elektronik, Lambrecht/Pfalz, Germany). Data acquisition was controlled with the WinWCP software (24). Data were sampled with 50 kHz and low-pass filtered with 10 kHz. Currents from oocytes were recorded in response to voltage steps of various amplitudes. During recordings of oocytes, extracellular superfusion of oocytes with HB was transiently switched to HB solutions containing 0.1, 1, 10, 100, or 500 μM Zn^{2+}.

VCF recordings

On the day of recording, oocytes were labeled at 4°C for 30 min in 0.05 mM (2-((5(6)-tetramethyl-rhodamine)carboxylamino)ethyl)methanethiosulfonate (MTS-TAMRA) dissolved in the HB recording solution. Following labeling, oocytes were washed three times in HB and stored at 18°C until recording. Subsequently, a single oocyte was placed in the recording chamber with the dark pole facing the top for VCF recordings (25,26). VCF recordings were performed with a BX51 upright microscope (Olympus, Tokyo, Japan) equipped with a water-immersion XLUMPlanFI objective (20×, NA 0.95, Olympus). The surface of the oocyte was excited with green light from an LED (LED4E099, Thorlabs, Newton, NJ). The LED was triggered by a TTL signal controlled by the WinWCP software. Light was passed through a Cy3 ET filter cube (excitation 545/25 nm, dichroic 565 nm LP, emission 605/70 nm; AHF Analysentechnik, Tübingen, Germany) and detected by a photo-diode (SM05PD2B, Thorlabs). The current from the photodiode was amplified with an Axopatch 200B amplifier (Molecular Devices, Union City, CA) and filtered with 10 kHz. Recordings were done in the C240S background. During recordings of each oocyte, extracellular superfusion of oocytes was transiently switched to solutions where 100 μM of Zn^{2+} was added.

Patch-clamp recordings

For excised inside-out patch-clamp recordings, a Multiclamp 700B amplifier (Molecular Devices) connected to a Digidata 1440A acquisition board (Molecular Devices) controlled by the ClampEx software (Molecular Devices) was used. Data were sampled with 50 kHz and low-pass filtered with 10 kHz. Recordings were performed under visual control using an Axiovert 200 upright microscope (Zeiss, Oberkochen, Germany). During recordings, the excised patch was superfused using a gravity-driven, TTL-controlled perfusion system ALA-VM8 (ALA Scientific instruments, Farmingdale, NY) to change from the EGTA-containing solution to the identical but EGTA-lacking solutions supplemented with 0.01, 0.1, 1, 10, or 100 μM Zn^{2+}. Leak currents were subtracted offline. *X. laevis* oocytes endogenously express Ca^{2+}-activated chloride channels (TMEM16A) (27), which are activated by low concentrations of free Ca^{2+} that are present in EGTA-free solutions. These chloride currents are voltage dependent but exhibit ohmic behavior and are smaller at negative membrane potentials. To minimize contamination of chloride currents in our recordings we chose a negative holding potential of -40 mV.

Data analysis

Data were analyzed with WinWCP, ClampFit (Molecular Devices), Excel (Microsoft, Redmond, WA), and/or Igor Pro (Wavemetrics, Portland, OR). The numbers of individual electrophysiological recordings are given as n_{oocyte} (TEVC and VCF recordings) or n_{patch} (patch-clamp recordings). Patches were excised from separate oocytes. Except for experiments displayed in the supporting figures, each experiment was performed on oocytes stemming from at least two different frogs. The concentration-response curves (Figs. 1, 5–7, and S2) were fit individually for each cell or patch from the relative inhibition with the Hill equation: $\%Inh_{max}/\left(1+\left(\frac{IC_{50}}{Zn^{2+}\ conc.}\right)^h\right)$, where $\%Inh_{max}$ is the maximal relative inhibition, IC_{50} is the half-maximal inhibition, and h is the Hill coefficient. The obtained mean parameters were used to construct the displayed fit. Activation and deactivation current kinetics were fit with a double-exponential function: $I = A_{fast}e^{\frac{-t}{\tau_{fast}}} + A_{slow}e^{\frac{-t}{\tau_{slow}}}$, where I is the current, A_{fast} and A_{slow} are the amplitudes, and τ_{fast} and τ_{slow} are the time constants for the fast and slow components, respectively. For the activation kinetics, the first 15 ms after stimulus onset showed a lag phase followed by a sigmoidal rise and were excluded from the fit. Fluorescence kinetics during channel activation in the VCF recordings was also fit with a double-exponential function to capture the rising and decaying components (Fig. 4). Current traces were boxcar filtered with a window width of 0.1 or 0.22 ms for displaying purpose. Fluorescence traces were boxcar filtered with a window width of 0.22 or 0.66 ms for displaying purpose. Statistical tests were performed with Igor Pro. The structural model of an HCNL1 subunit is from AlphaFold (28) and was rendered with PyMOL (29).

RESULTS

To study the effect of Zn^{2+} on HCNL1, we heterologously expressed HCNL1 in *X. laevis* oocytes and performed TEVC recordings (Fig. 1, *A* and *B*). Two to 5 h before recording, oocytes were injected with 50 nL of 1 M HEPES solution to increase the oocyte's pH buffer capacity to stabilize the intracellular pH during proton flux over the membrane (7). In addition, recordings were performed in extracellular solutions containing 100 mM HEPES (see materials and methods). A hyperpolarizing voltage step gave rise to an HCNL1-mediated inward current (Fig. 1 *B*). Application of Zn^{2+} reduced the inward current in a

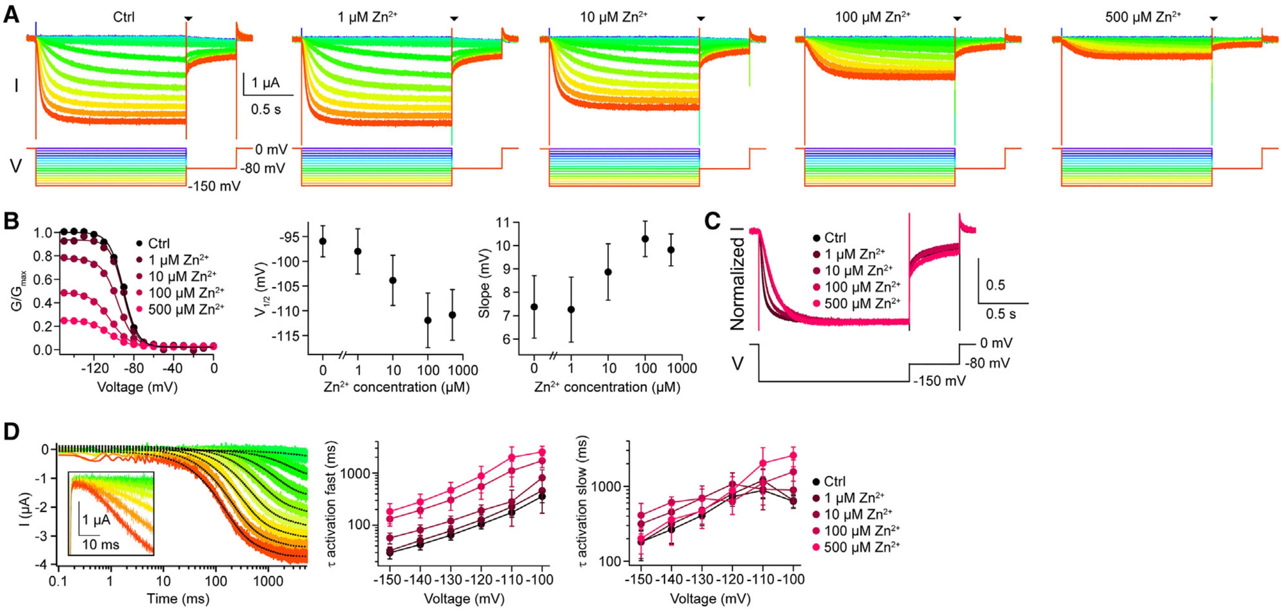

FIGURE 2 Zn^{2+} shifts the voltage dependence of activation and slows channel activation. (*A*) Representative HCNL1-mediated inward currents in response to a family of hyperpolarizing voltage steps for different Zn^{2+} concentrations. (*B*) Left: conductance-voltage relationships for different Zn^{2+} concentrations, derived from the tail currents of the data of (*A*) at the time points indicated by the triangle. Solid lines are Boltzmann fits. Middle and right: $V_{1/2}$ and slope of the Boltzmann fits of the current-voltage relationships ($n_{oocytes} = 6$). (*C*) HCNL1-mediated inward currents in response to a hyperpolarizing voltage step to -150 mV for various Zn^{2+} concentrations as in (*A*), but normalized to the steady-steady current amplitude. (*D*) Left: representative double-exponential fits (*dotted black traces*) of the activation kinetics of the inward current in the absence of Zn^{2+}. Inset: initial lag phase of approximately 10 ms at the beginning of activation that was excluded from fitting. Middle and right: voltage dependence of the fast and slow activation time constants for various Zn^{2+} concentrations ($n_{oocytes} = 6$). Data are represented as the mean $\pm$ SD.

concentration-dependent fashion (Fig. 1, *B* and *C*). We measured the relative inhibition for five different Zn^{2+} concentrations (Fig. 1 *D*). The resulting concentration-response relationship was fit with the Hill equation, revealing an apparent IC_{50} of 26 $\pm$ 10.3 μM (mean $\pm$ SD) and a Hill coefficient h of 0.78 $\pm$ 0.03 ($n_{oocytes} = 10$). The extrapolated maximal inhibition was 87.2 $\pm$ 7.1%. Washout of Zn^{2+} resulted in a recovery of the HCNL1-mediated inward current. However, recovery was not complete and reached on average 92 $\pm$ 5% of the initial amplitude.

Next, we investigated the mechanism by which Zn^{2+} inhibits HCNL1. Typical mechanisms of ion-channel inhibition are a classic pore block that inhibits conduction by steric occlusion of the pore, a shift of voltage dependence of the channel, or allosteric modulation of channel gating (1). Because voltage sensing, gating, and ion permeation are all located within the VSD of HCNL1, it can be anticipated that Zn^{2+} might inhibit HCNL1 by multiple mechanisms. We applied families of voltage steps to HCNL1-expressing oocytes in the presence of various extracellular Zn^{2+} concentrations (Fig. 2 *A*). From the tail currents, conductance-voltage relationships were obtained and Boltzmann fits revealed the voltage of half-maximal channel activation ($V_{1/2}$) and the slope (Fig. 2 *B*). Zn^{2+} (500 μM) shifted $V_{1/2}$ by -14.9 mV (control, $V_{1/2} = -95.9 \pm 3.1$ mV; 500 μM Zn^{2+}, $V_{1/2} = -110.8 \pm 5.1$ mV; $n_{oocytes} = 6$) to more negative membrane potentials and the slope by 2.4 mV (control, slope = 7.4 $\pm$ 1.3 mV; 500 μM Zn^{2+}, slope = 9.8 $\pm$ 0.7 mV). These data suggest that Zn^{2+} inhibits HCNL1 by shifting the channel's voltage dependence, e.g., by impeding channel opening, thus modulating channel gating. However, the Zn^{2+}-induced shift in voltage dependence of HCNL1 is by far not large enough to fully explain the inhibition efficacy of 81% of 500 μM at -120 mV, suggesting that Zn^{2+} inhibits HCNL1 currents by yet another mechanism, e.g., inhibiting the open state such as in a classical pore block mechanism. The activation kinetics of the HCNL1 currents are slowed by Zn^{2+}, as can be readily seen in normalized currents in response to a hyperpolarizing step to -150 mV at various Zn^{2+} concentrations (Fig. 2 *C*). The time course of activation is complex, with a lagging phase of approximately 10 ms at the beginning of the hyperpolarizing step (Fig. 2 *D*, *inset*), followed by an initial sigmoidal time course that is similar to the time course described for other VGICs, including Shaker (30,31), Hv1 (32,33), and HCN channels (17). This complex time course suggests that the HCNL1 channel undergoes multiple conformational changes before opening. The later kinetic components could be fit with a double-exponential function, yielding $\tau_{activation\ fast}$ and $\tau_{activation\ slow}$ (Fig. 2 *D*; see also materials and methods; $n_{oocytes} = 6$). Zn^{2+} slowed down $\tau_{activation\ fast}$ in a concentration-dependent manner at all tested voltages by 6- to 11-fold. No such clear impact of Zn^{2+} on $\tau_{activation\ slow}$ could be observed in our data.

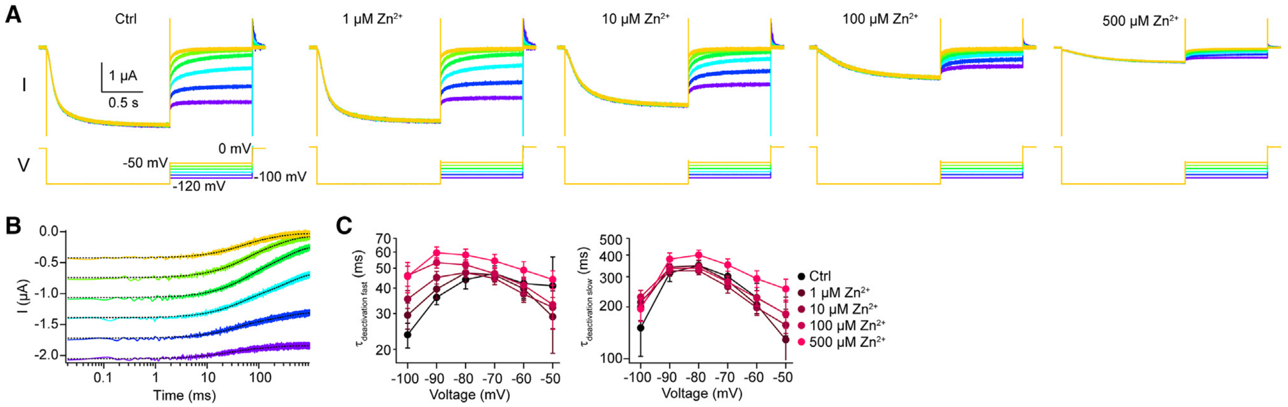

FIGURE 3 Zn^{2+} slows channel deactivation. (*A*) Representative HCNL1-mediated inward currents at various holding potentials following a hyperpolarizing voltage step to −120 mV in the presence or absence of Zn^{2+}. (*B*) Representative double-exponential fits (*dotted black traces*) of the deactivation kinetics of the inward current in the absence of Zn^{2+}. (*C*) Voltage dependence of the fast (*left*) and slow (*right*) deactivation time constants for various Zn^{2+} concentrations ($n_{\text{oocytes}} = 6$). Data are represented as the mean ± SD.

Next, we investigated the effect of Zn^{2+} and the deactivation kinetics (Fig. 3). To this end, HCNL1 was activated by a voltage step to −120 mV, followed by a step back to different voltages in the presence of various Zn^{2+} concentrations (Fig. 3 *A*). No initial lag phase was detected; the deactivation kinetics could be fit with a double-exponential function, yielding $\tau_{\text{deactivation fast}}$ and $\tau_{\text{deactivation slow}}$ (Fig. 3, *B* and *C*; $n_{\text{oocytes}} = 6$). Zn^{2+} slowed down $\tau_{\text{deactivation fast}}$ in a concentration-dependent manner at very negative voltages (−100, −90, and −80 mV); at less negative voltages, the effect of Zn^{2+} is less clear (Fig. 3 *C*). No clear Zn^{2+}-dependence of $\tau_{\text{deactivation slow}}$ could be observed in our data (Fig. 3 *C*). Taken together, our data are consistent with the idea that HCNL1 current inhibition by extracellular Zn^{2+} is due to multiple mechanisms, including inhibition of the open state and modulation of channel gating.

If the gating process of HCNL1 is modulated by Zn^{2+}, the underlying conformational changes should be altered by Zn^{2+}. We tested this prediction by employing VCF, which allows monitoring conformational changes in real time and in parallel to TEVC recordings (25,26). In brief, voltage-induced conformational changes of the ion-channel protein can be monitored as changes of the fluorescence of an environmentally sensitive fluorophore (e.g., TAMRA-MTS) that is attached to an introduced cysteine at the extracellular site of the channel of interest (Fig. 4 *A*). We screened for labeling sites in the C240S background at the extracellular end of S4 (J.K., unpublished data) and identified a site at position K163 that yielded large voltage-induced changes in TAMRA-MTS fluorescence (Fig. 4 *B*). Of note, the hyperpolarization-induced currents of the TAMRA-labeled HCNL1-K163C-C240S channel differ from the wild-type (WT) channel in voltage-dependence, kinetics, and Zn^{2+} inhibition: e.g., at −120 mV, the current of the TAMRA-labeled mutant is inhibited by 100 μM Zn^{2+} by only 30.1 ± 9.5% ($n_{\text{oocytes}} = 6$). The fluorescence change in response to an activating hyperpolarization to −120 mV displayed a complex kinetic behavior: a brief initial reduction in fluorescence (F_{initial}), a subsequent increase in fluorescence (F_{peak}), followed by fluorescence decline relaxing to a steady fluorescence intensity (F_{steady}) (Fig. 4 *C*, *black traces*). Repolarization (i.e., deactivation) elicited a transient decrease in fluorescence before relaxing back to the initial baseline fluorescence intensity; of note, a similar transient decrease during deactivation has been also observed in VCF recordings of Hv1 and has been termed F_{hook} (7,34,35). Zn^{2+} profoundly alters the voltage-induced changes in fluorescence during HCNL1 activation (Fig. 4 *C*, *magenta traces*): the amplitudes of F_{initial}, F_{peak}, and F_{steady} significantly changed when applying 100 μM Zn^{2+} (Fig. 4 *D*; $F_{\text{initial control}} = -0.18 \pm 0.07\%$, $F_{\text{initial Zn}} = -0.21 \pm 0.07\%$; $p = 0.028$; $F_{\text{peak control}} = 0.65 \pm 0.19\%$, $F_{\text{peak Zn}} = 1.01 \pm 0.32\%$, $p = 0.003$; $F_{\text{steady Control}} = -0.58 \pm 0.32\%$, $F_{\text{steady Zn}} = 0.53 \pm 0.23\%$, $p = 0.0009$; $n_{\text{oocytes}} = 6$, paired *t*-tests). The change in F_{steady} is most profound: the decrease in fluorescence in the absence of Zn^{2+} was inverted by Zn^{2+}, suggesting that a substantial fraction of channels reside in a distinct state in the presence of Zn^{2+}. We further analyzed the time course of the fluorescence changes during activation. While the signal/noise ratio of F_{initial} was too poor to resolve kinetics, the time course of the two later components (F_{peak} and F_{steady}) could be fit with a double-exponential function (Fig. 4 *C*, *dotted traces*). Zn^{2+} did not significantly change amplitude and time constant of the fast, rising component of the fluorescence change ($A_{\text{rise control}} = -1.55 \pm 0.45$, $A_{\text{rise Zn}} = -1.49 \pm 0.51$, $p = 0.46$; $\tau_{\text{rise control}} = 57.2 \pm 9.4$ ms, $\tau_{\text{rise Zn}} = 59.2 \pm 9.9$ ms, $p = 0.4$; paired *t*-tests). However, Zn^{2+} significantly changed amplitude and time constant of the slow, decaying component of the fluorescence change ($A_{\text{decay control}} = 2.09 \pm 0.79$, $A_{\text{decay Zn}} = 0.93 \pm 0.43$, $p = 0.0006$; $\tau_{\text{decay control}} = 293 \pm 34$ ms, $\tau_{\text{decay Zn}} = 379 \pm 52$ ms, $p = 0.03$; paired *t*-tests). Taken together, our fluorometry data suggest that inhibition of HCNL1

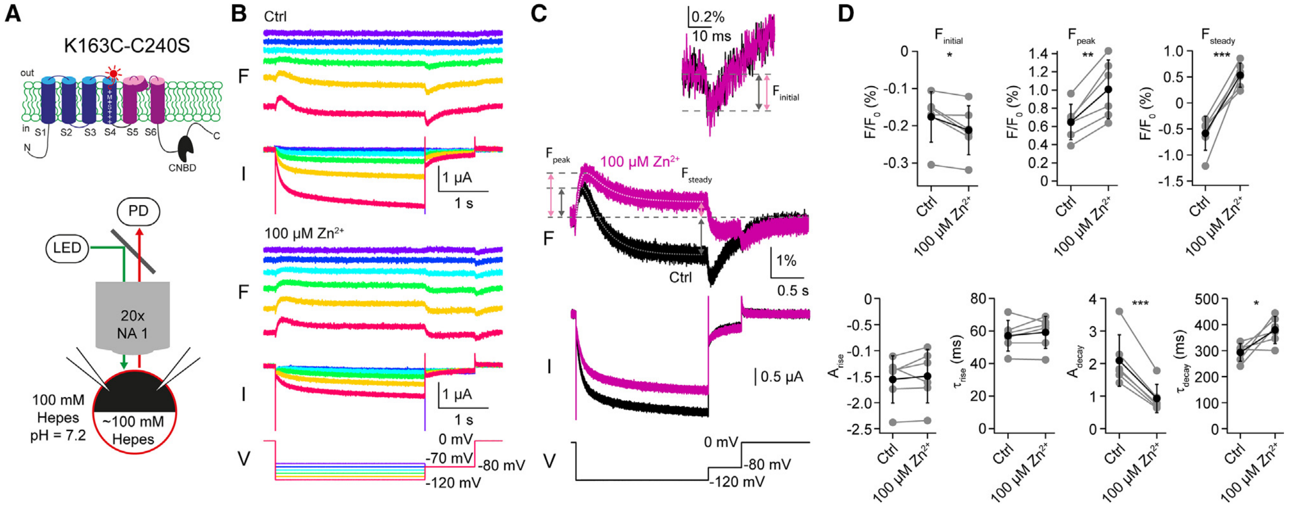

FIGURE 4 Zn^{2+} alters the voltage-induced conformational changes of the VSD. (*A*) Top: cartoon of one HCNL1 subunit indicating the cysteine at the extracellular end of transmembrane segment S4 (position 163) serving as an attachment site of the environmentally sensitive fluorophore TAMRA. Bottom: cartoon depicting the VCF recording configuration. (*B*) Representative fluorescence changes (F) and inward currents (I) in response to a family of hyperpolarizing voltage steps (*bottom*) in the absence (*top*) or presence of 100 μM Zn^{2+} (*middle*). Fluorescence traces are spread out for better visibility. (*C*) Representative fluorescence changes and currents in response to a hyperpolarizing voltage step in the presence (*magenta*) or absence (*black*) of 100 μM Zn^{2+}. Dotted lines are the double-exponential fits of the fluorescence traces during the hyperpolarizing voltage step. Amplitudes of F_{Peak} and F_{Steady} are indicated by arrows. Top: zoom-in of the initial reduction of fluorescence at the beginning of the hyperpolarizing voltage step. Amplitudes of $F_{initial}$ are indicated by arrows. (*D*) Top: amplitudes of the fluorescence signals $F_{initial}$, F_{Peak}, and F_{Steady} in the absence and presence of 100 μM Zn^{2+}. Bottom: parameters of the double-exponential fits of the fluorescence traces during the hyperpolarizing voltage step in the absence and presence of 100 μM Zn^{2+} ($n_{oocytes}$ = 6). Gray circles represent individual data points, black circles represent mean values; error bars denote SD.

current by Zn^{2+} involves allosteric modulation of the gating process by Zn^{2+} binding to the channel, resulting in a distinct state in the presence of Zn^{2+}.

Next, we aimed to identify the HCNL1 channel's binding site for Zn^{2+}. Zn^{2+} coordination sites in proteins are commonly provided by side chains of Cys, His, Asp, or Glu (36). In human Hv1, two His residues at the extracellular end of S2 (H140) and at the S3-S4 (H193) are important for Zn^{2+} inhibition; mutating both His residues to Ala virtually abolishes the channel's sensitivity to Zn^{2+} (2). Interestingly, HCNL1 also harbors two His residues at the extracellular face of the VSD: H78 and H82, with predicted location at the extracellular S1-S2 linker (AlphaFold, (28)) (Fig. 5 *A*). We tested the role of these residues in Zn^{2+} inhibition by mutagenesis. TEVC recordings of *Xenopus* oocytes expressing either HCNL1-H78A or HCNL1-H82A revealed only a modestly higher IC_{50} for Zn^{2+} (Fig. 5, *B–D*; H78A, IC_{50} = 55.9 ± 5.3 μM, $n_{oocytes}$ = 4; H82A, IC_{50} = 72.1 ± 28.3 μM, $n_{oocytes}$ = 6), suggesting that the His residues are either only weakly contributing to Zn^{2+} coordination or that other amino acid side chains compensate by providing alternative Zn^{2+} coordination.

Finally, we tested whether Zn^{2+} inhibits HCNL1 also from the intracellular side using excised inside-out patch-clamp recordings (Fig. 6 *A*). Macropatches of oocytes expressing HCNL1 were recorded in solutions at pH 7 containing 5 mM EGTA as a control or various concentrations of Zn^{2+} in the absence of EGTA. HCNL1 was activated by a voltage step from −40 to −120 mV. To our surprise, Zn^{2+} inhibited the HCNL1 current at lower concentrations from the intracellular than from the extracellular site (Fig. 6 *B*). The apparent IC_{50} was 0.53 ± 0.13 μM (Hill coefficient h = 0.97 ± 0.32, maximal inhibition 89.6 ± 6.8%, $n_{patches}$ = 4; mean values for 0.01 and 0.1 μM Zn^{2+} conditions are from 3 patches only). Interestingly, HCNL1 also harbors two His residues at the intracellular face of the VSD: H43 and H50, with predicted locations in the cytosol and at the intracellular end of S1, respectively (Fig. 7 *A*). We tested a role of these residues in Zn^{2+} inhibition by His to Ala mutagenesis and, analogous to WT HCNL1, subsequent excised inside-out patch-clamp recordings. Mutant HCNL1-H43A is inhibited by Zn^{2+} with a similar apparent IC_{50} (0.6 ± 0.4 μM) as WT (Fig. 7, *B* and *C*). In contrast, HCNL1-H50A is inhibited by Zn^{2+} only by much higher Zn^{2+} concentrations; the apparent IC_{50} is 106 ± 15 μM (Fig. 7, *B* and *C*). These data suggest that H50 but not H43 is important for intracellular Zn^{2+} binding and the current inhibition by intracellular Zn^{2+}.

The presence of an intracellular high affinity binding site for Zn^{2+} in combination with the slow and incomplete recovery of HCNL1 currents following washout of extracellular Zn^{2+} application (Fig. 1 *C*) may suggest that extracellular Zn^{2+} might enter the oocyte during the TEVC recording and exert its inhibition also (or even exclusively) from the intracellular site. Therefore, we tested whether Zn^{2+} permeates the HCNL1 channel in excised outside-out patch-clamp recordings by measuring the reversal potentials in the absence and presence of

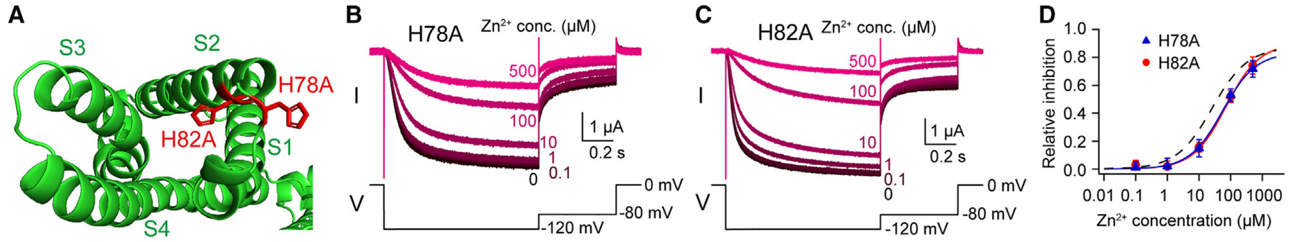

FIGURE 5 Histidine residues at positions 78 and 82 have limited contribution to Zn^{2+} binding. (*A*) Structural model of HCNL1 by AlphaFold (28). The cartoon shows the VSD from the extracellular side, perpendicular to the membrane plane. Amino acid side chains of histidine residues at positions 78 and 82 are shown and highlighted in red. (*B*) Representative inward currents of the H78A mutant in response to a hyperpolarizing voltage step in the presence or absence of various Zn^{2+} concentrations applied to the recording chamber. (*C*) Representative inward currents of the H82A mutant in response to a hyperpolarizing voltage step in the presence or absence of various Zn^{2+} concentrations applied to the recording chamber. (*D*) Concentration-response curves of the H78A ($n_{oocytes} = 4$) and H82A ($n_{oocytes} = 6$) mutants determined from the relative current inhibitions. The concentration-response curve of WT (same data as in Fig. 1 *D*) is depicted as a gray dashed curve for comparison. Data are represented as the mean ± SD.

extracellular Zn^{2+} (10 μM, Fig. S1 *A*). If Zn^{2+} permeated HCNL1, the reversal potential V_{rev} should deviate from the Nernst potential for protons ($E_{H+} = -59.2$ mV at intra- and extracellular pH of 6 and 7, respectively) by a large right-shift toward a less negative (or even positive) membrane potential in the presence of extracellular Zn^{2+}. However, we do not observe such a shift (Fig. S1, *A* and *B*). Alternatively, Zn^{2+} might enter by other means into the oocyte (leak or endogenous Zn^{2+} transporters). Therefore, we tested whether the HCNL1-H50A mutant, which is rather insensitive to intracellular Zn^{2+} (Fig. 7 *C*), is still inhibited by extracellular Zn^{2+}. This is indeed the case (Fig. S2, *A*–*C*); the apparent IC_{50} of HCNL1-H50A (7.3 ± 1.1 μM, $n_{oocytes} = 6$) was even slightly lower than the one of WT. We conclude that inhibition of HCNL1 currents by extracellular Zn^{2+} is not mediated by the intracellular Zn^{2+}-binding site. The incomplete current recovery might rather be due to difficulties in washing Zn^{2+} out of the TEVC recording chamber entirely; Zn^{2+} might be still around at low concentrations in the space between the vitelline membrane and the plasma membrane of the oocyte. Consistent with this idea, Zn^{2+} washout with a solution containing EGTA is more complete and faster than without EGTA (Fig. S3).

DISCUSSION

Several ion channels are modulated by low concentrations of Zn^{2+} (37). Of note, the voltage-gated proton channel Hv1 is inhibited by Zn^{2+} (2,3,38) and Otopetrin proton channels are inhibited (39) or activated by Zn^{2+} (40). Here, we investigated the effect of Zn^{2+} on the recently identified voltage-gated proton channel HCNL1. We found that HCNL1 currents are inhibited by extra- and intracellular application of Zn^{2+}. We characterized the extracellular inhibition by Zn^{2+} and suggest that inhibition is mediated by multiple mechanisms, including modulation of channel gating.

Classically, the mechanism of channel inhibition by small molecules or ions is described in categories such as pore blocking, shifting the voltage dependence, and modulation of voltage sensing or gating (1). The mechanisms are not mutually exclusive; one inhibitor can affect an ion channel by more than one mechanism. Because voltage sensing, gating, and ion permeation presumable all happen within the VSD of HCNL1, it is conceivable that Zn^{2+} inhibits via multiple mechanisms and influences various biophysical parameters. Indeed, extracellular Zn^{2+} affects HCNL1 currents in multiple ways: the voltage dependence of activation is shifted to more negative values (Fig. 2), the activation and deactivation kinetics are slowed (Figs. 2 and 3), the maximal conductance is reduced, and voltage-induced conformational changes are altered in the presence of Zn^{2+} (Fig. 4). This suggests that Zn^{2+} inhibits HCNL1 by multiple mechanisms. Of note, inhibitors of voltage-gated proton channels do not necessarily inhibit by multiple ways: 2GBI, an inhibitor of Hv1 and HCNL1, has been shown to act as an open channel pore blocker of Hv1, primarily occluding the permeation pathway for protons in the intracellular cavity of the VSD of Hv1 (41). The mechanism of HCNL1 current inhibition by 2GBI, however, has not been investigated in detail.

The proton channels HCNL1 and Hv1 share several common features. Both channels are voltage activated, conduct protons with high selectivity, and contain the ion-permeation pathway in their domains homologous to VSDs. It is worth comparing the mechanism of Zn^{2+} inhibition of the two channels. Similar to inhibition of HCNL1, inhibition of Hv1 by extracellular Zn^{2+} is also multifaceted: Zn^{2+} decreases the maximal conductance, shifts the conductance-voltage relationship to larger voltage excursions, and slows the activation time constant of Hv1 (15). Interestingly, slowing of the activation time constant seems to be a dominant effect of Zn^{2+} on Hv1 current inhibition (15). In contrast, the effect of Zn^{2+} on the time constants of activation (and deactivation) does not seem to be as dominant in HCNL1 (Figs. 2 and 3): Zn^{2+} slowed only the fast component of activation and deactivation consistently, while the slow component did not change much in the presence of Zn^{2+}.

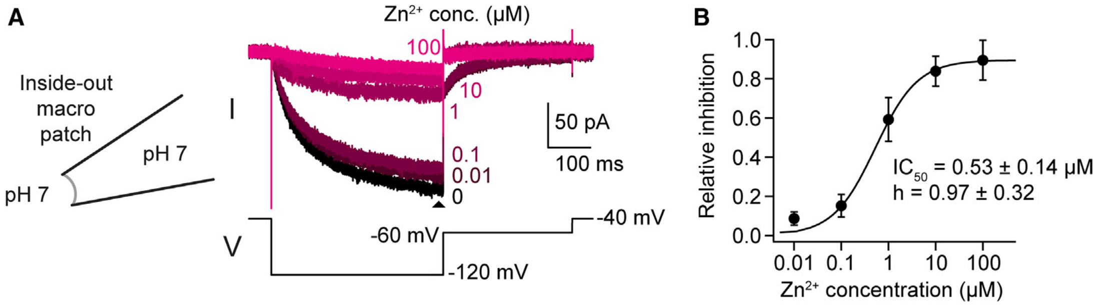

FIGURE 6 HCNL1 is inhibited by intracellular Zn^{2+}. (*A*) Representative current traces of an excised inside-out macropatch containing HCNL1 channels in response to hyperpolarizing voltage steps in the absence or presence of various Zn^{2+} concentrations. (*B*) Concentration-response curve determined from the relative current inhibition ($n_{patches} = 4$; mean values for 0.01 and 0.1 μM Zn^{2+} conditions are from 3 patches only). Data are represented as the mean $\pm$ SD.

In Hv1, two conserved histidine residues, located at the extracellular end of the S2 segment and between the S3 and S4 segments, are important for binding and inhibition by extracellular Zn^{2+} (2,42). The crystal structure of the mHv1cc proton channel (43) revealed additional, acidic amino acid residues at the extracellular end of segment S1 that are involved in coordinating Zn^{2+}, possibly indirectly by a water-mediated interaction. A study investigating *Ciona intestinalis* Hv1 using VCF and molecular dynamics simulations predicted two Zn^{2+}-binding sites within a subunit (34). The high affinity site was suggested to prevent channel opening, whereas the low-affinity site prevents outward S4 movement, i.e., interfered with voltage sensing. A consequence of two binding sites was seen in the voltage-induced fluorescence signal, which showed differential changes at different Zn^{2+} concentrations. We did not observe those changes; however, we cannot exclude that multiple Zn^{2+}-binding sites exist. Our VCF data suggest that several voltage-induced conformational changes of HCNL1 are altered in the presence of Zn^{2+}. Possibly, Zn^{2+} modulates the open state of HCNL1, leading to a lowered conductance.

Inhibition of Hv1 by intracellular Zn^{2+} is less well described but appears to be weak (15). Here, HCNL1 behaves differently; inhibition by Zn^{2+} from the intracellular site occurs at even lower concentrations than from the extracellular site (Fig. 6), with potential consequences for channel regulation under physiological conditions. It is unclear what the free Zn^{2+} concentration in sperm cytosol is and whether Zn^{2+} inhibition from this side of the channel is physiologically relevant. The total intracellular Zn^{2+} concentration has been estimated to be around 200–300 μM (44). Most intracellular Zn^{2+} is bound to proteins with high Zn^{2+}-binding affinity. Estimates for intracellular free Zn^{2+} vary depending on cell type and detection method; presumably, free intracellular Zn^{2+} is present at picomolar concentrations (44–46). Thus, intracellular Zn^{2+} could inhibit HCNL1 under physiological conditions only transiently when Zn^{2+} enters the sperm cell or is released from intracellular stores before Zn^{2+} is scavenged by Zn^{2+}-binding proteins with high affinity.

Several open questions remain regarding the mechanism of Zn^{2+} inhibition in HCNL1. Which amino acids coordinate Zn^{2+} at the intra- and extracellular sides of the HCNL1 channel? At the intracellular side, His at position 50 is involved in Zn^{2+} binding: The mutant HCNL1-H50A shows a profound shift of the IC_{50} by more than two orders of magnitude to higher Zn^{2+} concentrations. At the extracellular side, the His residues at positions 78 and 82 may weakly contribute to Zn^{2+} coordination. Investigating the Zn^{2+} sensitivity of double and triple mutants might be helpful to further delineate the residues that are important for binding Zn^{2+} in HCNL1. Additional residues must contribute to Zn^{2+} binding. High-affinity binding sites for Zn^{2+} in proteins, e.g., many zinc fingers, often coordinate Zn^{2+} with a tetrahedral geometry (47). Because HCNL1 is by several orders of magnitude less sensitive to Zn^{2+} as classical Zn^{2+}-binding proteins, it can be speculated that the Zn^{2+}-binding sites of HCNL1 do not follow the strict "standard" Zn^{2+} coordination geometry. Several charged amino acid side chains located at the extra- and intracellular ends are putative candidates for Zn^{2+} coordination. Standard mutagenesis approaches, e.g., replacing His by Ala residues and testing the mutant's inhibition by Zn^{2+}, can yield insights about the Zn^{2+}-binding site. However, functional compensation and even long-range effects by mutations at the opposite side of the membrane have been reported for Hv1 (42). Therefore, results from mutants need to be interpreted with caution. Is Zn^{2+} inhibition of HCNL1 currents pH dependent? In Hv1, acidification reduces Zn^{2+} inhibition, suggesting that protons and Zn^{2+} compete for the same binding sites (15). The effect of changes in pH on Zn^{2+} inhibition on either side of the HCNL1 remains to be addressed. Do other heavy metals inhibit HCNL1? While Zn^{2+} might be the only physiologically relevant divalent cation inhibiting HCNL1 currents, other heavy metals present in the environment might be burden for spawning zebrafish sperm by interfering with HCNL1 channel activity. For example, Hv1 is also inhibited by Cd^{2+} (15). Clearly, more research is needed to get a better understanding of the mechanism of the Zn^{2+}-mediated inhibition of HCNL1.

We previously showed that HCNL1 is expressed in zebrafish sperm and that its activity acidifies the intracellular pH

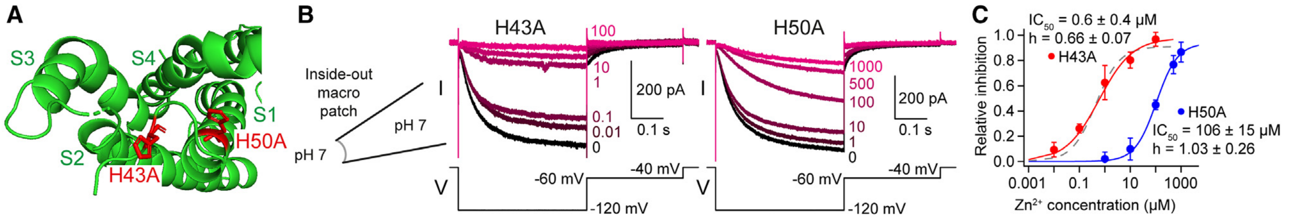

FIGURE 7 The histidine at position 50 is important for inhibition by intracellular Zn^{2+}. (*A*) Structural model of HCNL1 by AlphaFold. The cartoon shows the VSD from the intracellular side, perpendicular to the membrane plane. Amino acid side chains of histidine residues at positions 43 and 50 are shown and highlighted in red. (*B*) Representative current traces of excised inside-out macropatches containing HCNL1-H43A or HCNL1-H50A channels in response to hyperpolarizing voltage steps in the absence or presence of various Zn^{2+} concentrations. (*C*) Concentration-response curves determined from the relative current inhibition (HCNL1-H43A, $n_{\text{patches}} = 4$; HCNL1-H50A, $n_{\text{patches}} = 4$). The concentration response of WT (same data as in Fig. 6 *B*) is depicted as a gray dashed curve for comparison. Data are represented as the mean ± SD.

(16). Zn^{2+} has been suggested to play an important role in human sperm physiology. Early studies reported extraordinarily high concentrations of Zn^{2+} in seminal plasma, which could inhibit Hv1 in sperm following ejaculation (48). It has been suggested that dilution of Zn^{2+} in the female reproductive tract releases Zn^{2+} inhibition from Hv1 (14). Together with the more alkaline environment in the oviduct, Hv1 might contribute to sperm alkalization, which in turn promotes CatSper opening and Ca^{2+} influx and modulates flagellar beating, as well as acrosome reaction (49). Clinical studies suggest that Zn^{2+} is an important trace element for sperm health, and infertile males show significantly lower Zn^{2+} concentrations in seminal plasma (50). Interestingly, Zn^{2+} is an important trace element for spermatogenesis in the Japanese eel (51). However, caution has to be applied before generalizing findings in one species to another, in particular in the field of sperm physiology where species-specific differences and specializations are common (52). Whether Zn^{2+} plays a physiological role in zebrafish sperm physiology is not known. Yet, Zn^{2+} binding to HCNL1, which is expressed in the plasma membrane of the sperm head, suggests a relevance of Zn^{2+} in zebrafish sperm physiology. HCNL1 is activated by hyperpolarization, which occurs during spawning when sperm are released into freshwater: we previously suggested that the low K^{+} concentration in freshwater triggers a hyperpolarization due to K^{+} efflux via the CNGK channel (53), which in turn activates HCNL1. HCNL1 activation curtails hyperpolarization and acidifies the sperm cytosol (16), which in turn might trigger subsequent signaling events. HCNL1 activation could be prevented in the testis by Zn^{2+} inhibition if the ambient Zn^{2+} concentrations were high enough. It is difficult but would be useful to obtain an estimate of the concentrations of free Zn^{2+} in testis and milt of zebrafish (and of course sperm cytosol) to determine the putative physiological relevance of Zn^{2+} inhibition of HCNL1.

ACKNOWLEDGMENTS

We thank Dr. Olaf Pinkenburg and Irina Bogun for technical assistance and the members of the Department of Neurophysiology for support. M.F.K. is supported by a scholarship of the Deutscher Akademischer Austauschdienst.

AUTHOR CONTRIBUTIONS

D.O. and T.K.B. designed the research. M.F.K., J.K., J.M., E.D.M., and T.K.B. performed the research. M.F.K., J.K., E.D.M., and T.K.B. analyzed the data. M.F.K. and T.K.B. wrote the manuscript.

DECLARATION OF INTERESTS

The authors do not declare any conflicts of interest.

SUPPORTING MATERIAL

Supporting material can be found online at https://doi.org/10.1016/j.bpj.2024.08.018.

REFERENCES

1. Hille, B. 2001. Ion Channels of Excitable Membranes, 3rd edition. Sinauers Associates Inc.
2. Ramsey, I. S., M. M. Moran, …, D. E. Clapham. 2006. A voltage-gated proton-selective channel lacking the pore domain. *Nature.* 440:1213–1216.
3. Sasaki, M., M. Takagi, and Y. Okamura. 2006. A voltage sensor-domain protein is a voltage-gated proton channel. *Science.* 312:589–592.
4. Koch, H. P., T. Kurokawa, …, H. P. Larsson. 2008. Multimeric nature of voltage-gated proton channels. *Proc. Natl. Acad. Sci. USA.* 105:9111–9116.
5. Lee, S. Y., J. A. Letts, and R. MacKinnon. 2008. Dimeric subunit stoichiometry of the human voltage-dependent proton channel Hv1. *Proc. Natl. Acad. Sci. USA.* 105:7692–7695.
6. Tombola, F., M. H. Ulbrich, and E. Y. Isacoff. 2008. The voltage-gated proton channel Hv1 has two pores, each controlled by one voltage sensor. *Neuron.* 58:546–556.
7. Gonzalez, C., H. P. Koch, …, H. P. Larsson. 2010. Strong cooperativity between subunits in voltage-gated proton channels. *Nat. Struct. Mol. Biol.* 17:51–56.
8. Tombola, F., M. H. Ulbrich, …, E. Y. Isacoff. 2010. The opening of the two pores of the Hv1 voltage-gated proton channel is tuned by cooperativity. *Nat. Struct. Mol. Biol.* 17:44–50.
9. DeCoursey, T. E. 2003. Voltage-gated proton channels and other proton transfer pathways. *Physiol. Rev.* 83:475–579.

10. Bennett, A. L., and I. S. Ramsey. 2017. CrossTalk opposing view: proton transfer in Hv1 utilizes a water wire, and does not require transient protonation of a conserved aspartate in the S1 transmembrane helix. *J. Physiol.* 595:6797–6799.
11. DeCoursey, T. E. 2017. CrossTalk proposal: Proton permeation through Hv1 requires transient protonation of a conserved aspartate in the S1 transmembrane helix. *J. Physiol.* 595:6793–6795.
12. DeCoursey, T. E., and V. V. Cherny. 1993. Potential, pH, and arachidonate gate hydrogen ion currents in human neutrophils. *Biophys. J.* 65:1590–1598.
13. Iovannisci, D., B. Illek, and H. Fischer. 2010. Function of the HVCN1 proton channel in airway epithelia and a naturally occurring mutation, M91T. *J. Gen. Physiol.* 136:35–46.
14. Lishko, P. V., I. L. Botchkina, …, Y. Kirichok. 2010. Acid extrusion from human spermatozoa is mediated by flagellar voltage-gated proton channel. *Cell.* 140:327–337.
15. Cherny, V. V., and T. E. DeCoursey. 1999. pH-dependent inhibition of voltage-gated H(+) currents in rat alveolar epithelial cells by Zn(2+) and other divalent cations. *J. Gen. Physiol.* 114:819–838.
16. Wobig, L., T. Wolfenstetter, …, T. K. Berger. 2020. A family of hyperpolarization-activated channels selective for protons. *Proc. Natl. Acad. Sci. USA.* 117:13783–13791.
17. Gauss, R., R. Seifert, and U. B. Kaupp. 1998. Molecular identification of a hyperpolarization-activated channel in sea urchin sperm. *Nature.* 393:583–587.
18. Ludwig, A., X. Zong, …, M. Biel. 1998. A family of hyperpolarization-activated mammalian cation channels. *Nature.* 393:587–591.
19. Santoro, B., D. T. Liu, …, G. R. Tibbs. 1998. Identification of a gene encoding a hyperpolarization-activated pacemaker channel of brain. *Cell.* 93:717–729.
20. De La Rosa, V., and I. S. Ramsey. 2018. Gating Currents in the Hv1 Proton Channel. *Biophys. J.* 114:2844–2854.
21. Carmona, E. M., M. Fernandez, …, C. Gonzalez. 2021. The voltage sensor is responsible for ΔpH dependence in Hv1 channels. *Proc. Natl. Acad. Sci. USA.* 118:e2025556118.
22. Ramsey, I. S., Y. Mokrab, …, D. E. Clapham. 2010. An aqueous H+ permeation pathway in the voltage-gated proton channel Hv1. *Nat. Struct. Mol. Biol.* 17:869–875.
23. Cherny, V. V., V. S. Markin, and T. E. DeCoursey. 1995. The voltage-activated hydrogen ion conductance in rat alveolar epithelial cells is determined by the pH gradient. *J. Gen. Physiol.* 105:861–896.
24. Dempster, J. 1997. A new version of the Strathclyde Electrophysiology software package running within the Microsoft Windows environment. *J. Physiol.* 504:P57.
25. Cha, A., and F. Bezanilla. 1997. Characterizing voltage-dependent conformational changes in the Shaker K+ channel with fluorescence. *Neuron.* 19:1127–1140.
26. Mannuzzu, L. M., M. M. Moronne, and E. Y. Isacoff. 1996. Direct physical measure of conformational rearrangement underlying potassium channel gating. *Science.* 271:213–216.
27. Schroeder, B. C., T. Cheng, …, L. Y. Jan. 2008. Expression cloning of TMEM16A as a calcium-activated chloride channel subunit. *Cell.* 134:1019–1029.
28. Jumper, J., R. Evans, …, D. Hassabis. 2021. Highly accurate protein structure prediction with AlphaFold. *Nature.* 596:583–589.
29. Schrödinger, LLC. 2015. The PyMOL molecular graphics system, version 1.8.
30. Hoshi, T., W. N. Zagotta, and R. W. Aldrich. 1994. Shaker potassium channel gating. i: Transitions near the open state. *J. Gen. Physiol.* 103:249–278.
31. Zagotta, W. N., T. Hoshi, …, R. W. Aldrich. 1994. Shaker potassium channel gating. II: Transitions in the activation pathway. *J. Gen. Physiol.* 103:279–319.
32. Villalba-Galea, C. A. 2014. Hv1 proton channel opening is preceded by a voltage-independent transition. *Biophys. J.* 107:1564–1572.
33. DeCoursey, T. E., and V. V. Cherny. 1994. Voltage-activated hydrogen ion currents. *J. Membr. Biol.* 141:203–223.
34. Qiu, F., A. Chamberlin, …, H. P. Larsson. 2016. Molecular mechanism of Zn2+ inhibition of a voltage-gated proton channel. *Proc. Natl. Acad. Sci. USA.* 113:E5962–E5971.
35. Qiu, F., S. Rebolledo, …, H. P. Larsson. 2013. Subunit interactions during cooperative opening of voltage-gated proton channels. *Neuron.* 77:288–298.
36. Laitaoja, M., J. Valjakka, and J. Jänis. 2013. Zinc coordination spheres in protein structures. *Inorg. Chem.* 52:10983–10991.
37. Peralta, F. A., and J. P. Huidobro-Toro. 2016. Zinc as allosteric ion channel modulator: Ionotropic receptors as metalloproteins. *Int. J. Mol. Sci.* 17:1059.
38. Mahaut-Smith, M. P. 1989. The effect of zinc on calcium and hydrogen ion currents in intact snail neurones. *J. Exp. Biol.* 145:455–464.
39. Tu, Y. H., A. J. Cooper, …, E. R. Liman. 2018. An evolutionarily conserved gene family encodes proton-selective ion channels. *Science.* 359:1047–1050.
40. Teng, B., J. P. Kaplan, …, E. R. Liman. 2022. Structural motifs for subtype-specific pH-sensitive gating of vertebrate otopetrin proton channels. *Elife.* 11:e77946.
41. Hong, L., M. M. Pathak, …, F. Tombola. 2013. Voltage-sensing domain of voltage-gated proton channel Hv1 shares mechanism of block with pore domains. *Neuron.* 77:274–287.
42. De La Rosa, V., A. L. Bennett, and I. S. Ramsey. 2018. Coupling between an electrostatic network and the Zn2+-binding site modulates Hv1 activation. *J. Gen. Physiol.* 150:863–881.
43. Takeshita, K., S. Sakata, …, A. Nakagawa. 2014. X-ray crystal structure of voltage-gated proton channel. *Nat. Struct. Mol. Biol.* 21:352–357.
44. Maret, W. 2015. Analyzing free zinc (ii) ion concentrations in cell biology with fluorescent chelating molecules. *Metallomics.* 7:202–211.
45. Bozym, R. A., R. B. Thompson, …, C. A. Fierke. 2006. Measuring picomolar intracellular exchangeable zinc in PC-12 cells using a ratiometric fluorescence biosensor. *ACS Chem. Biol.* 1:103–111.
46. Maret, W., and Y. Li. 2009. Coordination dynamics of zinc in proteins. *Chem. Rev.* 109:4682–4707.
47. Neuhaus, D. 2022. Zinc finger structure determination by nmr: Why zinc fingers can be a handful. *Prog. Nucl. Magn. Reson. Spectrosc.* 130–131:62–105.
48. Saaranen, M., U. Suistomaa, …, T. Vanha-Perttula. 1987. Lead, magnesium, selenium and zinc in human seminal fluid: comparison with semen parameters and fertility. *Hum. Reprod.* 2:475–479.
49. Zhao, R., K. Kennedy, …, S. A. N. Goldstein. 2018. Role of human Hv1 channels in sperm capacitation and white blood cell respiratory burst established by a designed peptide inhibitor. *Proc. Natl. Acad. Sci. USA.* 115:E11847–E11856.
50. Zhao, J., X. Dong, …, L. Li. 2016. Zinc levels in seminal plasma and their correlation with male infertility: A systematic review and meta-analysis. *Sci. Rep.* 6:22386.
51. Yamaguchi, S., C. Miura, …, T. Miura. 2009. Zinc is an essential trace element for spermatogenesis. *Proc. Natl. Acad. Sci. USA.* 106:10859–10864.
52. Kaupp, U. B., and T. Strünker. 2017. Signaling in sperm: More different than similar. *Trends Cell Biol.* 27:101–109.
53. Fechner, S., L. Alvarez, …, U. B. Kaupp. 2015. A K(+)-selective CNG channel orchestrates Ca(2+) signalling in zebrafish sperm. *Elife.* 4:e07624.

Biophysical Journal, Volume *123*

Supplemental information

Zinc inhibits the voltage-gated proton channel HCNL1

Makoto F. Kuwabara, Joschua Klemptner, Julia Muth, Emilia De Martino, Dominik Oliver, and Thomas K. Berger

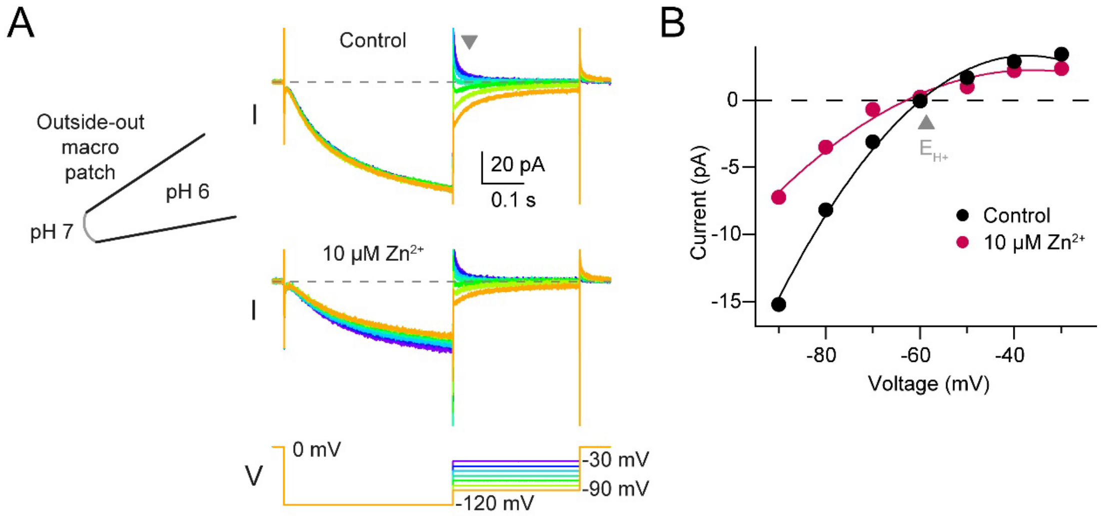

FIGURE S1. Zn^{2+} does not permeate through HCNL1. (A) Current traces of an excised outside-out macro patch containing HCNL1 channels in response to hyperpolarizing voltage steps in the absence (Control) or presence of 10 µM Zn^{2+} applied to the bath. (B) Current–voltage relationships of the tail currents in panel (A). E_{H+} is the Nernst potential for protons (-59.2 mV at 25° C and intra- and extracellular pH of 6 and 7, respectively), indicated by the gray triangle. If HCNL1 conducted Zn^{2+}, the tail current would reverse its sign at less negative potentials. Instead, there is a slight shift towards more negative potentials (control, V_{rev} = -62.3 ± 1.6 mV; 10 µM Zn^{2+}, V_{rev} = -64.6 ± 1.2 mV, $n_{patches}$ = 4). Zeros crossing were determined by fitting the data to quadratic functions.

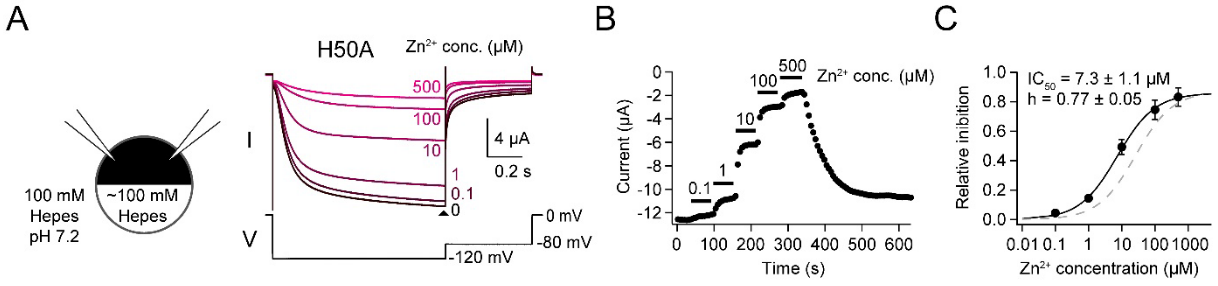

FIGURE S2. Similar to WT, HCNL1-H50A is inhibited by extracellular Zn^{2+}. (A) Left, cartoon of the TEVC recording configuration. Prior to recording, the pH buffer capacity was increased by an injection of HEPES. Right, representative HCNL1-H50A-mediated inward currents in response to a hyperpolarizing voltage step in the presence or absence of various Zn^{2+} concentrations applied to the recording chamber. (B) Steady-state current amplitudes during Zn^{2+} application, derived from the data of panel (A) at the time point indicated by the triangle. (C) Concentration-response curve determined from the relative current inhibition ($n_{oocytes}$ = 6). The extrapolated maximal inhibition was 85.9 ± 6.0%. The concentration response of WT (same data as in Figure 1D) is depicted as a gray dashed curve for comparison. Data are represented as the mean ± SD.

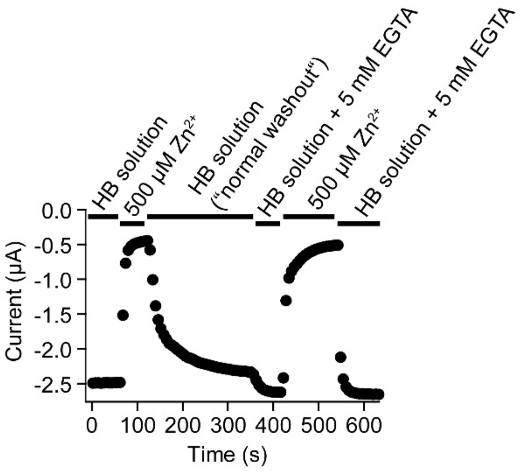

FIGURE S3. Extracellular application of EGTA improves current recovery after Zn^{2+} washout. Steady-state current amplitudes of an HCNL1-expressing oocyte before and after extracellular application of 500 µM Zn^{2+} in the absence ("normal washout") or presence of 5 mM EGTA during washout. Current recovery after washout of Zn^{2+} was 89.7 ± 6.8% in the absence of EGTA and 99.6 ± 1.7% in the presence of EGTA ($n_{Oocytes}$ = 6).
